# A Novel Nonenzymatic Hydrogen Peroxide Sensor Based on Magnetic Core-Shell Fe_3_O_4_@C/Au Nanoparticle Nanocomposite

**DOI:** 10.1155/2021/8839895

**Published:** 2021-03-08

**Authors:** Xiao Ni, Mingwei Tian, Jun Sun, Xiaojun Chen

**Affiliations:** College of Chemistry and Molecular Engineering, Nanjing Tech University, Nanjing 211800, China

## Abstract

Fe_3_O_4_@C/Au nanoparticle (AuNP) nanocomposites were prepared through electrostatic adsorption of AuNPs onto PDDA-functionalized core/shell Fe_3_O_4_@C magnetic nanospheres, which had been synthesized by a facile solvothermal method. The morphology and composition of the nanocomposites were characterized by transmission electron microscopy (TEM), scanning electron microscopy (SEM), Fourier transform infrared (FT-IR), etc. Moreover, highly electrocatalytic activity to the reduction of hydrogen peroxide (H_2_O_2_) was also exhibited on the Fe_3_O_4_@C/AuNP-modified indium tin oxide (ITO) electrode. The effect of solution pH and the modification amount of Fe_3_O_4_@C/AuNPs on the performance of electrocatalytic H_2_O_2_ reduction was investigated. Under the optimal conditions, the catalytic current showed a linear relationship with the increase of H_2_O_2_ concentration in the range of 0.007–15 mM and a detection limit of 5 *μ*M. The H_2_O_2_ sensor showed high selectivity for H_2_O_2_ detection, which could effectively resist the interference of ascorbic acid (AA), uric acid (UA), and citric acid (CA). Finally, the H_2_O_2_ sensor was used in the real fetal bovine serum to detect H_2_O_2_ and obtained satisfactory results with the recovery values ranging from 95.14 to 103.6%.

## 1. Introduction

Magnetic nanoparticles have been popularly applied in anode material [1], degradation [2], electromagnetic wave absorption [3], separation [4], and catalysis [5]. Among these diverse magnetic nanomaterials reported before, Fe_3_O_4_ NPs have been studied extensively because of their excellent magnetism, biocompatibility, and especially, highly effective electrocatalytic activity. For example, Kingsley et al. fabricated Fe_3_O_4_ nanoparticle- (NP-) modified carbon paste electrode to detect ascorbic acid (AA) and folic acid (FA) at the same time, with the detection limits of 2.51 × 10^–7^ and 2.01 × 10^–9^ M, respectively [6]. Qu and coworkers established a novel hydrogen peroxide (H_2_O_2_) sensor using Fe_3_O_4_ NPs/chitosan composite-modified electrode, which could electrocatalyze the reduction of H_2_O_2_ with a detection limit of 1.53 × 10^–6^ M [7]. However, Fe_3_O_4_ NPs are unstable and prone to be aggregated and settled in aqueous solution because of the magnetic dipolar-dipolar attraction, resulting in the decrease of their catalytic activity [8]. To overcome this problem, considerable efforts have been devoted to introduce stabilizers on the surface of Fe_3_O_4_ NPs.

In recent years, core/shell structured magnetic nanocomposites have been proposed, with Fe_3_O_4_ NPs as the core and inorganic materials or polymers as the shell. These specific core/shell Fe_3_O_4_ materials possess enhanced stability and compatibility with different charges, functions, or reactive moieties. Owing to the enlarged effective surface area and the synergetic interaction between core and shell, these nanocomposites usually combine the advantageous properties of both core and shell [9]. For example, Beitollahi and coworkers synthesized a carbon paste electrode modified with magnetic Fe_3_O_4_@SiO_2_/MWCNT nanocomposite and found it exhibiting a very good resolution between the voltammetric peaks of hydrazine and phenol, which made it suitable for the detection of hydrazine in the presence of phenol in real samples [10]. Wang and coworkers showed that the Fe_3_O_4_@ZnO nanoparticles (NPs) have excellent photocatalytic activity and it is convenient to reuse NPs in wastewater treatment owing to the magnetic Fe_3_O_4_ core [11]. Therefore, magnetic core/shell nanocomposites with multiple properties have been widely used.

Among these shell materials, carbon and gold are the most applied coating materials due to their excellent conductivity and biocompatibility [12, 13]. In addition, on the surface of carbon shells, abundant amounts of hydroxyl (-OH) and carboxyl (-COOH) groups can effectively protect the Fe_3_O_4_ NPs from aggregation and facilitate further functional modification over the surface. Hence, the applicability of Fe_3_O_4_@C has been extensively promoted. For example, Zhang and coworkers synthesized Fe_3_O_4_@C NPs, which showed dramatic ability to remove methylene blue, with a maximum adsorption of 141.3 mg·g^−1^ [14]. Zhang and coworkers fabricated another Fe_3_O_4_@C NPs immobilized upon the GCE which was modified with molecular imprinted TiO_2_ velamen. It was then made into the uric acid sensor, which delivered a linear range of 0.3–34 *μ*M with a detection limit of 0.02 *μ*M [15]. Meanwhile, functionalized Fe_3_O_4_@Au NPs have also been applied in As (III) [16] and dopamine [17] sensors, respectively.

In this work, Fe_3_O_4_@C core/shell nanocomposite was synthesized by a facile solvothermal method from ferrocene as the raw material. It was not only easily separated by an external magnetic field but also possessed excellent dispersion in water. To further enhance the conductivity and catalytic activity, Au NPs were adsorbed on the surface of the obtained Fe_3_O_4_@C nanospheres by electrostatic attraction. The synthesized Fe_3_O_4_@C/Au NPs were first used to fabricate a nonenzymatic H_2_O_2_ sensor and showed high electrochemical sensitivity.

## 2. Materials and Methods

### 2.1. Materials

Ferrocene, acetone, sodium citrate, absolute ethanol, sodium chloride (NaCl), and Tris base were purchased from Shanghai Lingfeng Chemical Reagent (China). Poly(diallyldimethylammonium chloride) (PDDA, 20 wt. %) was purchased from Aldrich. Chloroauric acid hydrated (HAuCl_4_·4H_2_O) and sodium citrate were purchased from Nanjing Chemical Reagent (China). H_2_O_2_ (30%) was obtained from Sinopharm Chemical Reagent. Phosphate buffer saline (PBS, 0.1 M) was prepared by mixing the stock solutions of KH_2_PO_4_ and K_2_HPO_4_ and was adjusted to the appropriate pH value. Uric acid (UA), AA, and citric acid (CA) were all purchased from Sigma. Nafion (Nf) was obtained from DuPont as 5 wt% solution and was used as 0.5 wt% solution after dilution with water. Other reagents were of analytical grade and used without further purification. Double distilled water (DDW) was used throughout the experiments.

### 2.2. Characterization

The structures and morphologies of the Fe_3_O_4_@C and Fe_3_O_4_@C/AuNPs were characterized by transmission electron microscopy (TEM, JEOL JEM-200CX) and field emission scanning electron microscopy (FESEM, Hitachi S4800). Fourier transform infrared (FT-IR) spectra of KBr powder-pressed pellets were recorded on a Bruker model VECTOR 22 Fourier transform spectrometer. Elemental composition and chemical valence analyses were performed on X-ray diffraction (XRD, Rigaku SmartLab) and X-ray photoelectron spectroscopy (XPS, Thermo Scientific K-Alpha), respectively.

Cyclic voltammetry (CV), differential pulse voltammetry (DPV), and electrochemical impedance spectroscopy (EIS) were performed using a CHI 660D electrochemical workstation (Shanghai Chenhua Instruments, China). A three-compartment electrochemical cell contained a saturated calomel reference electrode (SCE), a platinum wire auxiliary electrode, and a modified ITO working electrode with a 3 mm diameter controlled area. The determination of H_2_O_2_ was conducted in 0.1 M PBS, and the sensor responses were measured under deductions for background currents from the total ones.

### 2.3. Preparation of Fe_3_O_4_@C/AuNP Magnetic Nanocomposite

Core/shell structured Fe_3_O_4_@C magnetic nanocomposite was prepared by Wang's method [18]. First, 0.3 g of ferrocene was dissolved in 30 mL of acetone under ultrasonication at room temperature for 30 min. Then, 1 mL of H_2_O_2_ was added dropwise into the above ferrocene solution. After reacting for 15 min under stirring, the resulting solution was transferred into a Teflon-lined stainless-steel autoclave with a capacity of 50 mL and heated at 210°C for 24 h. After cooling to room temperature, the product was magnetically separated, washed several times with absolute ethanol, and finally dried under vacuum at 50°C for 5 h.

Au colloid solution was prepared by adding 5 mL of 1% (wt. %) sodium citrate into 200 mL of 0.01% (wt. %) HAuCl_4_, which had been heated to boil beforehand. Heating was stopped by the change of solution color from light blue to violet-red and stirring was continued for 15 min. After cooling to room temperature, Au colloid solution was preserved in a refrigerator at 4°C. Generally, an electrostatic attraction method was used for the immobilization of AuNPs onto the surface of the Fe_3_O_4_@C core/shell nanocomposite. 50 mg of Fe_3_O_4_@C nanocomposite was dispersed into 30 mL of 0.2% PDDA that contained 20 mM Tris base and 20 mM NaCl, and the mixture was stirred for 20 min. The product was collected with the help of a magnet and rinsed with DDW six times to remove the residual PDDA. The positively charged Fe_3_O_4_@C nanocomposite was dispersed in 100 mL of Au colloid solution and stirred for 8 h. The suspension was separated with the help of a magnet. After rinsed with water and ethanol three times successively, the Fe_3_O_4_@C/AuNPs nanocomposite was dried under vacuum at 50°C for 6 h.

### 2.4. Fabrication of Fe_3_O_4_@C/AuNP-Modified ITO Magnetism Electrode

Before modification, the ITO chips were cleaned with acetone, ethanol, and water, respectively, and then dried under a stream of nitrogen. The active electrode area was confined by bounding a circular opened 3M polyvinyl chloride (PVC) film on the ITO surface, with a 3 mm diameter on the middle of the electrode. On the other side of the ITO, a piece of 8 mm diameter and 5 mm height permanent magnet was adhered by silica rubber adhesive [19]. Then, 10 *µ*L of 1 mg·mL^−1^ Fe_3_O_4_@C/AuNP nanocomposite dispersion (in 5 mL 0.5% Nf) was dropped onto the surface of the ITO magnetism electrode and dried at room temperature, which was designated as Fe_3_O_4_@C/AuNPs/Nf/ITO. When the sensor was not in use, it was stored at 4°C in a refrigerator.

## 3. Results and Discussion

### 3.1. Structure and Composition Characterization of Samples

**Figure 1:**
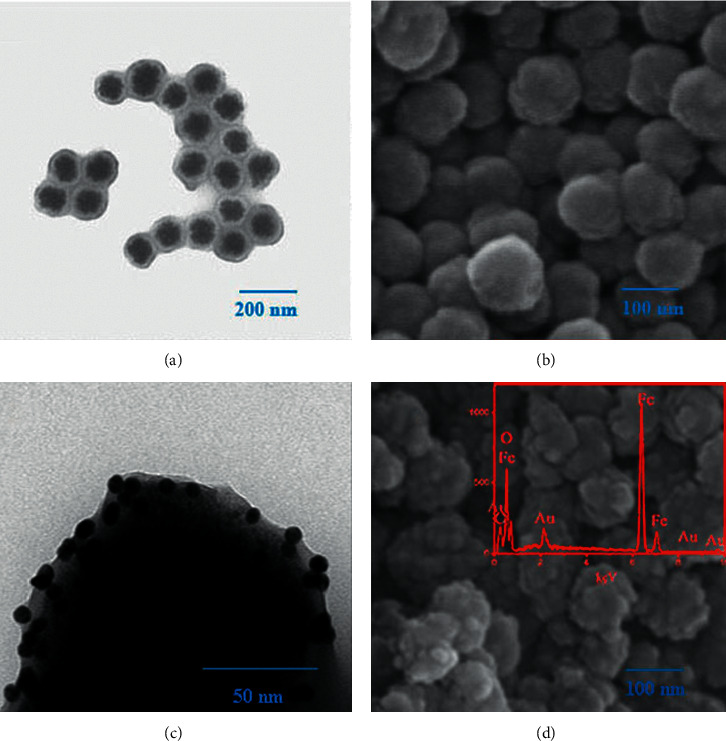
(a, c) TEM and (b, d) FESEM images of Fe_3_O_4_@C and Fe_3_O_4_@C/AuNPs. The inset shows the elemental composition of Fe_3_O_4_@C/AuNPs.

The structure and morphology of the nanocomposites were investigated by TEM and FESEM. In Figure 1(a), Fe_3_O_4_@C with obvious core/shell structures were successfully synthesized. The presence of carbon shells prevented agglomeration of the Fe_3_O_4_ NPs and improved the dispersity of the Fe_3_O_4_@C, with an average diameter of approximately 125 nm. As vividly shown in Figure 1(b), the SEM image of Fe_3_O_4_@C displays a distinct margin without agglomeration. Figure 1(c) shows the TEM image of Fe_3_O_4_@C/AuNPs with a diameter of 141 nm. It is clear that there are numerous dark spots embedded in the outer shells, indicating that AuNPs were successfully immobilized on the surface of the Fe_3_O_4_@C nanospheres, with an average diameter of approximately 15 nm. Figure 1(d) shows the SEM image of the rather rough surface of Fe_3_O_4_@C/AuNPs with a raspberry-shaped morphology compared to that of Fe_3_O_4_@C. Moreover, some of them are overlapped or amalgamated due to external immobilization of AuNPs, which amplify the superficial roughness. And the dispersity of the Fe_3_O_4_@C nanocomposites is weakened by electrostatic attraction between AuNPs and Fe_3_O_4_@C as well. The inset in Figure 1(d) shows the elemental composition of Fe_3_O_4_@C/AuNPs and the Au atom mass concentration is 3.57%.

**Figure 2:**
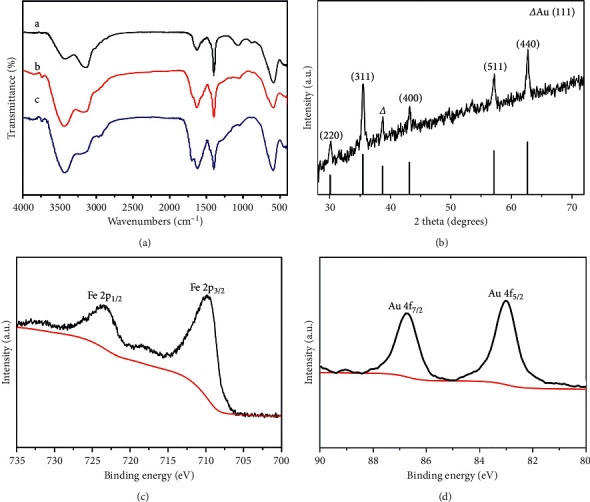
(a) FT-IR spectra of the Fe_3_O_4_ (curve a), Fe_3_O_4_@C (curve b), and Fe_3_O_4_@C/AuNPs (curve c), respectively, (b) XRD spectra of the Fe_3_O_4_@C/AuNPs. XPS spectra of (c) Fe 2p and (d) Au 4f from Fe_3_O_4_@C/AuNPs.

FT-IR was used to characterize the chemical structure of the samples.  Figure 2(a) shows the FT-IR spectra of Fe_3_O_4_ (curve a), Fe_3_O_4_@C (curve b), and Fe_3_O_4_@C/AuNPs (curve c). The characteristic peak appears at 590 cm^−1^ which is related to the Fe-O stretching vibration [20]. Compared with curve a, there is a new strong band in curve b at around 3446 cm^−1^, indicating the presence of –OH in the carbon shell. These phenomena further proved that the carbon shell is successfully coated on the surface of the Fe_3_O_4_ nanosphere. The peak located at 1399 cm^−1^ is attributed to the COO- symmetric vibration [21]. Actually, the carboxyl group-functionalized Fe_3_O_4_@C nanospheres are negatively charged. This is the reason why we use PDDA to make the surface of carbon shell positively charged for the assembly of negatively charged AuNPs via electrostatic interaction. Curve c shows the FT-IR spectrum of Fe_3_O_4_@C/AuNPs. Because AuNPs do not have absorption in the IR region, the characteristic peak of Fe_3_O_4_@C/AuNPs is almost the same as that of Fe_3_O_4_@C.

To characterize the crystal information of the prepared Fe_3_O_4_@C/AuNPs further, the XRD has been performed. As shown in Figure 2(b), the diffraction peaks appeared at 30°, 35.5°, 43°, 57°, and 62°are originated from Fe_3_O_4_, corresponding to (220), (311), (400), (511), and (440) crystal planes [22]. In addition to those peaks, another peak at 38.2° is in good accordance with (111) plane of AuNPs. It is difficult to discriminate Fe_3_O_4_ phases from *γ*-Fe_2_O_3_ by only using XRD patterns due to the similarity of patterns, and XPS is conducted to characterize the electronic structures of Fe element in the nanocomposite, which is known to be sensitive to Fe^2+^ and Fe^3+^ cations.  Figure 2(c) presents two peaks at the binding energies of 724.4 and 711.4 eV, which correspond to the maximum intensity of Fe 2p1/2 and Fe 2p3/2, illustrating the coexistence of Fe^2+^ and Fe^3+^ cations in Fe_3_O_4_@C/AuNPs [23]. Peaks of Au are also shown in Figure 2(d), corresponding to Au 4f2/7 and Au 4f2/5, respectively. From the above characterization results, the well-defined core/shell structured Fe_3_O_4_@C/AuNPs is confirmed to be constructed.

### 3.2. Characterization of Nanocomposite-Modified ITO Electrodes

**Figure 3:**
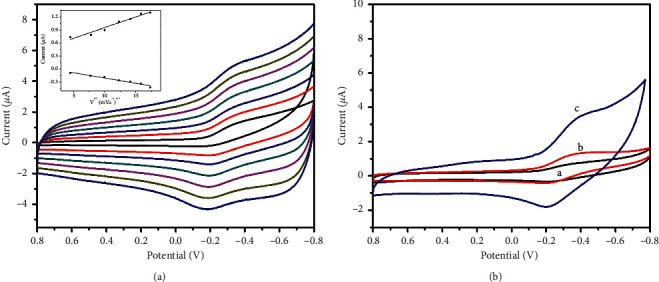
(a) CVs of Fe_3_O_4_@C/AuNPs/ITO at the scan rates of 40, 60, 100, 150, 200, 250, and 300 mV·s^−1^ (from internal to external) in 0.1 M pH 7.4 PBS containing 0.1 mM H_2_O_2_, respectively. The inset shows the linear plots of the peak current vs. *v*^1/2^; (b) CVs of different modified electrodes: bare ITO (curve a), Fe_3_O_4_@C/ITO (curve b), and Fe_3_O_4_@C/AuNPs/ITO (curve c) in 0.1 M pH 7.4 PBS containing 0.1 mM H_2_O_2_ at a scan rate of 100 mV·s^−1^.

The influence of potential scan rate (*v*) on the response of the Fe_3_O_4_@C/AuNP-modified ITO electrode in PBS was studied, as shown in Figure 3(a). The reduction and oxidation peak currents (i_pa_ and i_pc_) increased linearly with the square root of scan rate (*v*^1/2^) accelerating from 40 to 300 mV·s^−1^, indicating a surface-controlled electrochemical process.

As shown in Figure 3(b), we also recorded the CVs of various modified electrodes towards 0.1 mM H_2_O_2_ in 0.1 M pH 7.4 PBS at a scan rate of 100 mV·s^−1^. At the bare ITO, almost no peak of H_2_O_2_ is observed (curve a), except for a slight reduction peak. After the electrode was modified with Fe_3_O_4_@C nanocomposites, a small increase in i_pa_ was observed (curve b), indicating the electrocatalytic reduction activity toward H_2_O_2_ possessed by Fe_3_O_4_@C nanocomposites. The reduction peak potential was around −0.35 V, similar to another reported nonenzymatic H_2_O_2_ sensor [24] and the i_pa_ was 1.7 times higher compared with that in curve a. When the Fe_3_O_4_@C/AuNPs were assembled on the electrode, the peak current significantly increased (curve c) and the i_pa_ is 2.4 times that of curve b, demonstrating that the AuNPs significantly improve the catalytic activity of Fe_3_O_4_@C nanocomposites.

**Figure 4:**
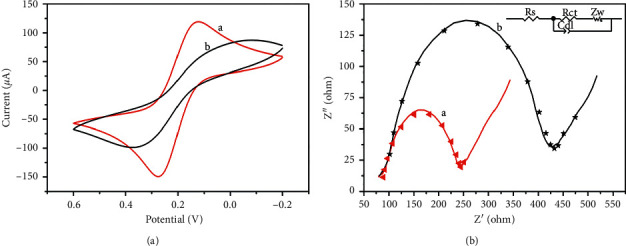
(a) CV and (b) EIS curves of Fe_3_O_4_@C/AuNPs/ITO (curve a) and Fe_3_O_4_@C/ITO (curve b) in 0.1 M of pH 7.4 PBS containing 0.1 M KCl and 5 mM [Fe(CN)6]^3−/4−^.

The electron transfer rate and the interfacial resistance, as shown in Figures 4(a) and 4(b), respectively, can reflect the electrochemical properties. In Figure 4(a), obvious oxidation and reduction peaks were observed on both Fe_3_O_4_@C- and Fe_3_O_4_@C/AuNP-modified electrodes, belonging to different valences of Fe element. An increased peak current was found after surface modification with AuNPs, indicating that noble metals could promote electron transfer on the electrode. In addition, the electroactive surface area (ESA) was calculated to be 1.628 cm^2^ by Randles–Sevcik equation, which is nearly 23 times the geometric area. It means that the modification of Fe_3_O_4_@C/AuNPs increased the roughness of the electrode surface greatly, which could facilitate the absorption, transmission, and catalysis of H_2_O_2_.

Similar results were also confirmed by EIS measurements, as shown in Figure 4(b). The diameter of the Nyquist curve revealed charge transfer resistance of the electrode (R_ct_), and a larger diameter means a greater resistance. Curve a presented a smaller R_ct_ value at Fe_3_O_4_@C/AuNP-modified ITO, verifying AuNPs could enhance charge transfer significantly, compared with that in curve b.

### 3.3. Optimization of Experimental Parameters

**Figure 5:**
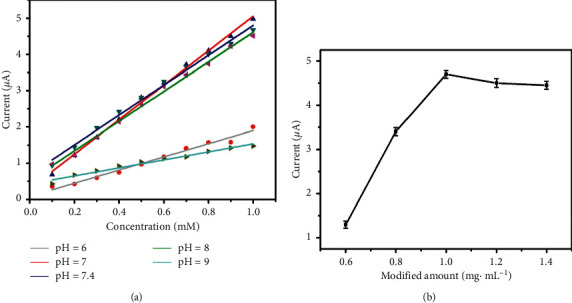
(a) Linear plots of the reduction peak current versus the concentration of H_2_O_2_ from 0.1 to 1 mM over the pH range from 6 to 9 at Fe_3_O_4_@C/AuNPs/ITO electrode; (b) dependence of reduction peak current of 1 mM H_2_O_2_ in 0.1 M pH 7.4 PBS upon the modification amount of Fe_3_O_4_@C/AuNPs.

The variations of oxidation-reduction potential of H_2_O_2_ in different pH values indicate diverse oxidation ability of H_2_O_2_, resulting in different sensitivities of sensors under different pH circumstances. The effect of solution pH on the electrocatalytic reduction of H_2_O_2_ was investigated.  Figure 5(a) shows the linear plots of the reduction peak current versus the concentration of H_2_O_2_ from 0.1 to 1 mM over the pH range from 6 to 9. The electrocatalytic activity and sensitivity were much higher at the pH value of 7–8 and lower at 6 and 8. The possible reason was that Fe_3_O_4_ core was expected to dissolve in acidic circumstances and the catalytic activity faded. Meanwhile, H_2_O_2_ is prone to lose oxidizing ability with solution alkalinity increasing. Therefore, a nearly neutral condition is suitable for H_2_O_2_ detection and the PBS with physiologic pH value of human being which is about 7.4 was chosen as the supporting electrolyte.

Figure 5(b) exhibits reduction peak current versus different modification amounts of Fe_3_O_4_@C/AuNPs. The response current towards 1 mM H_2_O_2_ rose along with the modification amount of Fe_3_O_4_@C/AuNPs increasing from 0.6 to 1 mg·mL^−1^ and reached a plateau when the modification amount exceeded 1 mg mL^−1^. The response speed was supposed to be delayed by thickening modification, which brought hidden troubles of its falling off from the electrode surface. It may lead to a decreased stability of the modified electrode and the smaller response current. In this way, 1 mg·mL^−1^ of Fe_3_O_4_@C/AuNPs was regarded to be the optimal amount of modification for H_2_O_2_ detection.

### 3.4. Amperometric Response of H_2_O_2_ Sensor

**Figure 6:**
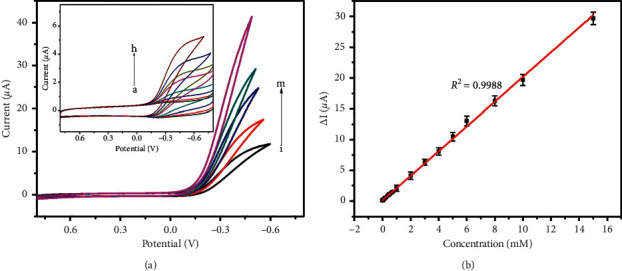
(a) CVs of Fe_3_O_4_@C/AuNPs/ITO recorded in 0.1 M pH 7.4 PBS containing 0.007–15 mM of H_2_O_2_ (curves a to m, at a scan rate of 100 mV·s^−1^. The inset shows the CVs corresponding to the H_2_O_2_ concentration in the range of 0.007–1 mM (from a to h); (b) the calibration plot of the reduction peak current vs. the H_2_O_2_ concentration.

Figure 6 displays CVs in the presence of H_2_O_2_ at different concentrations under the optimum conditions. The response current shows a good linear relationship with the increase of H_2_O_2_ concentration in the range of 0.007–15 mM. The linear regression equations is ∆i_p_ (*µ*A) = 0.10 + 2.01 *c* (H_2_O_2_) (mM) (*R*^2^ = 0.9988). The detection limit was estimated at 5 *µ*M, and the theoretical sensitivity of this sensor was calculated to be 28.71 *µ*A mM^−1^ cm^−2^. The response current became saturated when the concentration of H_2_O_2_ exceeded 15 mM. Quantitative detection of H_2_O_2_ concentration can be realized by the calculation with the linear equation. Furthermore, the performances including electrode modification, linear range, and detection limit of the Fe_3_O_4_@C/AuNPs/ITO were compared with those of other H_2_O_2_ sensors, which are listed in Table 1. It was shown that the proposed H_2_O_2_ sensor in this work exhibited a wider linear range and a relatively lower detection limit. In comparison with those previous works, the advantages of our work are ascribed to five aspects: (1) the combination of the magnetic core of Fe_3_O_4_, thin layer of C, and uniformly dispersed AuNP modification shows their synergies and thus enhances the catalytic ability towards H_2_O_2_; (2) the C layer prevents Fe_3_O_4_ catalyst from aggregating and maintains its catalytic activity; (3) the utilization of magnetic electrode increases the modification amount of Fe_3_O_4_@C/AuNPs and enhances the stability of the electrode modification; (4) the C layer is not compact, which facilitate H_2_O_2_ and the reaction products transferring between the bulk solution and electrode surface; and (5) the modification of AuNPs enhances the electron transfer and electrochemical activity of the H_2_O_2_ sensor.

### 3.5. Stability and Repeatability

**Figure 7:**
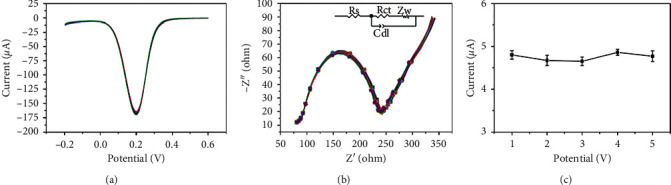
(a) DPV and (b) EIS curves of Fe_3_O_4_@C/AuNPs/ITO in 0.1 M of pH 7.4 PBS containing 0.1 M KCl and 5 mM [Fe(CN)6]^3-/4-^ with 10 continuous cycles of scanning. (c) The reduction peak current of 1 mM H_2_O_2_ in 0.1 M pH 7.4 PBS recorded on 5 parallel sensors.

The application of chemical sensors put forward higher demands on stability and catalytic signal repeatability. DPV and EIS measurements for Fe_3_O_4_@C/AuNP-modified ITO were conducted in 0.1 M of pH 7.4 PBS containing 0.1 M KCl and 5 mM [Fe(CN)6]^3-/4-^. As shown in Figure 7, it was found that the DPV peak remained nearly unchanged after 10 cycles of scanning. On the other hand, the Nyquist plots nearly overlapped for 10 times of detection, and the R_ct_ value maintained around 152 Ω. It demonstrated that the immobilized Fe_3_O_4_@C/AuNP modification layer could keep a good level of stability in a continuous scanning system.

In addition, a repeated measurement towards 1 mM H_2_O_2_ in 0.1 M pH 7.4 PBS was conducted to verify the repeatability of the sensor. The response signal recorded on 5 parallel electrodes is displayed in Figure 7(c), and the relative standard deviation (RSD) value between them was calculated as 1.8%. The good stability and repeatability of the fabricated H_2_O_2_ sensor are attributed to the simple modification method, stable property, and good catalytic ability of Fe_3_O_4_@C/AuNPs.

### 3.6. Interference Study

**Figure 8:**
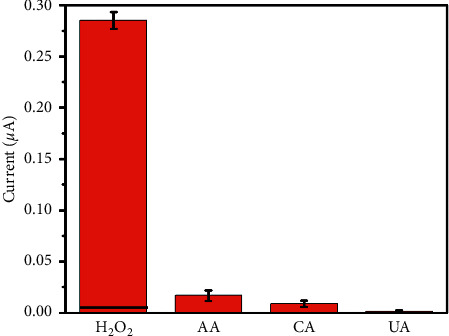
Current responses after addition of 0.1 mM of H_2_O_2_, AA, UA, and CA at the potential of −0.35 V, respectively.

To evaluate the selectivity of Fe_3_O_4_@C/AuNPs, an interference study was carried out. As it is known, AA, UA, and CA are the most common interfering electroactive species during the detection of H_2_O_2_. The current responses with the addition of H_2_O_2_, AA, UA, and CA at the potential of −0.35 V are shown in Figure 8, among which the responses caused by AA, UA, and CA interfering species were no more than 10% response of H_2_O_2_. The result implied that the Fe_3_O_4_@C/AuNPs possessed high selectivity for H_2_O_2_ detection at the potential of −0.35 V.

### 3.7. Determination of H_2_O_2_ in Fetal Bovine Serum (FBS)

The determination of H_2_O_2_ in FBS was also investigated. The FBS was diluted with 0.1 M of pH 7.4 PBS via the volume ratio of 1 : 9. We measured the recovery by adding different concentrations of H_2_O_2_ into the FBS dilution. The recovery values were calculated ranging from 95.14 to 103.6%, which are presented in Table 2. The relative standard deviations (RSDs) are less than 5%, proving a good application prospect.

## 4. Conclusion

In summary, a novel core-shell structured Fe_3_O_4_@C/AuNPs nanocomposite is successfully synthesized and utilized for fabricating a nonenzymatic H_2_O_2_ sensor, exhibiting a wider linear range from 0.007 to 15 mM and a lower detection limit of 5 *μ*M with a sensitivity of 28.71 *µ*A mM^−1^ cm^−2^. The advantages of the proposed sensor over the previous works are concluded as four points: (1) the superparamagnetic property of Fe_3_O_4_@C/AuNPs nanocomposite contributes to the construction of a magnetic electrode modification, enhancing the stability of the sensor greatly. (2) The protection of C/AuNPs bilayer ensures the monodispersity of Fe_3_O_4_ catalyst, maintaining its good catalytic activity for H_2_O_2_ reduction. (3) The C/AuNPs bilayer possesses hydrophilicity, biocompatibility, electroactivity, and conductivity, facilitating the substance transmission and electron transfer between bulk solution and electrode surface. (4) The detection of H_2_O_2_ in FBS illustrates high selectivity, displaying good potential for real applications. Of course, the fabricated Fe_3_O_4_@C/AuNPs nanocomposite could also be used as substrate or nanolabel in other sensing systems.

## Figures and Tables

**Table 1:** The performance comparison of different nonenzymatic H_2_O_2_ sensors.

Electrode modification	Linear range (mM)	Detection limit (*μ*M)	Ref.
Co_3_O_4_/MWCNTs	0.02–0.43	2.46	[25]
PtAu	0.05–1.15	4.1	[26]
Pd/CNT	0.008–9.5	2.6	[27]
AgCo/MWCNTs	0.05–10	0.74	[28]
NPG@Ni	0.02–9.74	10	[29]
Se/PtNPs	0.01–1.5	3.1	[30]
Ag/NaX	0.02–11.76	9.1	[31]
Ag NW array	0.1–3.1	29.2	[32]
AgNPs	0.05–6.5	27	[33]
Fe_3_O_4_@C/CuNPs	0.08–372	32.6	[34]
Fe_3_O_4_@C/AuNPs	0.007–15	5	This work

**Table 2:** The recovery of H_2_O_2_ by standard addition method in FBS samples.

Sample	Added concentration (mM)	Measured concentration (mM)	Recovery (%)
1	0.094	0.09	95.14
2	0.28	0.29	103.6
3	0.47	0.48	102.1
4	0.95	0.94	98.95
5	4.54	4.62	101.8

## Data Availability

The data that support the findings of this study are available from the corresponding author upon reasonable request.

## References

[1] Liang C.-L., Liu Y., Bao R.-Y. (2016). Effects of Fe_3_O_4_ loading on the cycling performance of Fe_3_O_4_/rGO composite anode material for lithium ion batteries. *Journal of Alloys and Compounds*.

[2] Aziz H. Y., Maryam S. G. (2016). Fe_3_O_4_/ZnO/Ag_3_VO_4_/AgI nanocomposites: quaternary magnetic photocatalysts with excellent activity in degradation of water pollutants under visible light. *Separation and Purification Technology*.

[3] Wang Y., Lai Y., Wang S., Jiang W. (2017). Controlled synthesis and electromagnetic wave absorption properties of core-shell Fe_3_O_4_@SiO_2_ nanospheres decorated graphene. *Ceramics International*.

[4] Muliwa A. M., Leswifi T. Y., Onyango M. S., Maity A. (2016). Magnetic adsorption separation (MAS) process: an alternative method of extracting Cr(VI) from aqueous solution using polypyrrole coated Fe_3_O_4_ nanocomposites. *Separation and Purification Technology*.

[5] Chang J., Ma Q., Ma J., Ma H. (2016). Synthesis of Fe_3_O_4_ nanowire@CeO_2_/Ag nanocomposites with enhanced photocatalytic activity under sunlight exposure. *Ceramics International*.

[6] Kingsley M. P., Desai P. B., Srivastava A. K. (2015). Simultaneous electro-catalytic oxidative determination of ascorbic acid and folic acid using Fe_3_O_4_ nanoparticles modified carbon paste electrode. *Journal of Electroanalytical Chemistry*.

[7] Qu J., Dong Y., Lou T., Du X. (2014). Determination of hydrogen peroxide using a novel sensor based on Fe_3_O_4_Magnetic nanoparticles. *Analytical Letters*.

[8] Xie J., Cao H., Jiang H. (2013). Co_3_O_4_-reduced graphene oxide nanocomposite as an effective peroxidase mimetic and its application in visual biosensing of glucose. *Analytica Chimica Acta*.

[9] Tian R., Chen X., Xu X., Yao C. (2014). Electrocatalytic activity of core/shell magnetic nanocomposite. *Analytical Biochemistry*.

[10] Beitollahi H., Tajik S., Jahani S. (2016). Electrocatalytic determination of hydrazine and phenol using a carbon paste electrode modified with Ionic Liquids and Magnetic Core-shell Fe_3_O_4_@SiO_2_/MWCNT Nanocomposite. *Electroanalysis*.

[11] Wang J., Yang J., Li X. (2016). Preparation and photocatalytic properties of magnetically reusable Fe_3_O_4_@ZnO core/shell nanoparticles. *Physica E: Low-Dimensional Systems and Nanostructures*.

[12] Orhan A., Aykut C., Hilal K., Ozlem S. (2019). Dendrimer templated synthesis of carbon nanotube supported PdAu catalyst and its application as hydrogen peroxide sensor. *Electroanalysis.*.

[13] Hilal C. K., Firat S., Aykut C., Hilal K., Nahit A. (2018). Synthesis, characterization, and voltammetric hydrogen peroxide sensing on novel monometallic (Ag, Co/MWCNT) and bimetallic (AgCo/MWCNT) alloy nanoparticles. *Fullerenes, Nanotubes and Carbon Nanostructures*.

[14] Zhang W., Zhang L. Y., Zhao X. J., Zhou Z. (2016). Citrus pectin derived ultrasmall Fe_3_O_4_@C nanoparticles as a high-performance adsorbent toward removal of methylene blue. *Journal of Molecular Liquids*.

[15] Zhang C., Si S., Yang Z. (2015). A highly selective photoelectrochemical biosensor for uric acid based on core-shell Fe_3_O_4_@C nanoparticle and molecularly imprinted TiO_2_. *Biosensors and Bioelectronics*.

[16] Wei J., Li S.-S., Guo Z., Chen X., Liu J.-H., Huang X.-J. (2016). Adsorbent assisted in situ electrocatalysis: an ultra-sensitive detection of As(III) in water at Fe_3_O_4_ nanosphere densely decorated with Au nanoparticles. *Analytical Chemistry*.

[17] Qiu J.-D., Xiong M., Liang R.-P., Peng H.-P., Liu F. (2009). Synthesis and characterization of ferrocene modified Fe_3_O_4_@Au magnetic nanoparticles and its application. *Biosensors and Bioelectronics*.

[18] Wang H., Chen Q.-W., Yu Y.-F., Cheng K., Sun Y.-B. (2011). Size- and solvent-dependent magnetically responsive optical diffraction of carbon-encapsulated superparamagnetic colloidal photonic crystals. *The Journal of Physical Chemistry C*.

[19] Chen X., Zhu J., Chen Z., Xu C., Wang Y., Yao C. (2011). A novel bienzyme glucose biosensor based on three-layer Au-Fe_3_O_4_@SiO_2_ magnetic nanocomposite. *Sensors and Actuators B: Chemical*.

[20] Gao Q., Chen F., Zhang J. (2009). The study of novel Fe_3_O_4_@*γ*-Fe_2_O_3_ core/shell nanomaterials with improved properties. *Journal of Magnetism and Magnetic Materials*.

[21] Xuan S., Wang Y.-X. J., Yu J. C., Leung K. C.-F. (2009). Preparation, characterization, and catalytic activity of core/shell Fe_3_O_4_@polyaniline@Au nanocomposites. *Langmuir*.

[22] Gong J., Yao K., Liu J. (2013). Striking influence of Fe_2_O_3_ on the “catalytic carbonization” of chlorinated poly(vinyl chloride) into carbon microspheres with high performance in the photo-degradation of Congo red. *Journal of Materials Chemistry A*.

[23] Zhang J., Ji Y., Wang P., Shao Q., Li Y., Huang X. (2020). Adsorbing and activating N_2_ on heterogeneous Au-Fe_3_O_4_ nanoparticles for N_2_ fixation. *Advanced Functional Materials*.

[24] Liao K., Mao P., Li Y. (2013). A promising method for fabricating Ag nanoparticle modified nonenzyme hydrogen peroxide sensors. *Sensors and Actuators B: Chemical*.

[25] Heli H., Pishahang J. (2014). Cobalt oxide nanoparticles anchored to multiwalled carbon nanotubes: synthesis and application for enhanced electrocatalytic reaction and highly sensitive nonenzymatic detection of hydrogen peroxide. *Electrochimica Acta*.

[26] Wang J., Gao H., Sun F., Xu C. (2014). Nanoporous PtAu alloy as an electrochemical sensor for glucose and hydrogen peroxide. *Sensors and Actuators B: Chemical*.

[27] Hilal C. K., Aykut C., Tarik A., Nahit A., Hilal K. (2018). Microstructured prealloyed Titanium-Nickel powder as a novel nonenzymatic hydrogen peroxide sensor. *Journal of Colloid and Interface Science*.

[28] Hilal D. K., Tarik A., Aykut C. (2009). Electrochemical production of graphene oxide and its application as a novel hydrogen peroxide sensor. *International Journal of Nano Dimension*.

[29] Ke X., Li Z., Gan L. (2015). Three-dimensional nanoporous Au films as high-efficiency enzyme-free electrochemical sensors. *Electrochimica Acta*.

[30] Li Y., Zhang J.-J., Xuan J., Jiang L.-P., Zhu J.-J. (2010). Fabrication of a novel nonenzymatic hydrogen peroxide sensor based on Se/Pt nanocomposites. *Electrochemistry Communications*.

[31] Azizi S. N., Ghasemi S., Kavian S. (2014). Synthesis and characterization of NaX nanozeolite using stem sweep as silica source and application of Ag-modified nanozeolite in electrocatalytic reduction of H_2_O_2_. *Biosensors and Bioelectronics*.

[32] Kurowska E., Brzózka A., Jarosz M., Jaskuła G. D., Jaskuta M. (2013). Silver nanowire array sensor for sensitive and rapid detection of H_2_O_2_. *Electrochimica Acta*.

[33] Raoof J. B., Ojani R., Rashid-Nadimi E., Hasheminejad S., Rashid-Nadimi S. (2012). Electrochemical synthesis of Ag nanoparticles supported on glassy carbon electrode by means of p-isopropyl calix[6]arene matrix and its application for electrocatalytic reduction of H_2_O_2_. *Applied Surface Science*.

[34] Zhang M., Sheng Q., Nie F., Zheng J. (2014). Synthesis of Cu nanoparticles-loaded Fe_3_O_4_@carbon core-shell nanocomposite and its application for electrochemical sensing of hydrogen peroxide. *Journal of Electroanalytical Chemistry*.

